# Surgical Correction of Carcinoid Heart Disease Improves Liver Function and 5-Hydroxyindoleacetic Acid Levels

**DOI:** 10.3389/fsurg.2022.791058

**Published:** 2022-04-08

**Authors:** Husnain Abbas Shah, Vandana Sagar, Simon Hughes, Amardeep Khanna, Ivan Yim, Freya Lodge, Harjot Singh, Tessa Oelofse, Críostóir Ó'Súilleabháin, Hema Venkataraman, Shishir Shetty, Richard Steeds, Stephen Rooney, Tahir Shah

**Affiliations:** ^1^Department of Hepatology, Queen Elizabeth Hospital Birmingham, Birmingham, United Kingdom; ^2^Department of Imaging, Queen Elizabeth Hospital Birmingham, Birmingham, United Kingdom; ^3^Department of Cardiothoracic Surgery, Queen Elizabeth Hospital Birmingham, Birmingham, United Kingdom; ^4^Department of Cardiology, Queen Elizabeth Hospital Birmingham, Birmingham, United Kingdom; ^5^Featherstone Department of Anaesthesia and Intensive Care, Queen Elizabeth Hospital Birmingham, Birmingham, United Kingdom; ^6^Department of Hepatobiliary Surgery, Mercy University Hospital, Cork, Ireland; ^7^Department of Endocrinology, Queen Elizabeth Hospital Birmingham, Birmingham, United Kingdom

**Keywords:** carcinoid heart disease, 5-HIAA, congestive hepatopathy, valve replacement surgery, neuroendocrine tumors (NETs)

## Abstract

**Introduction:**

Carcinoid heart disease (CHD) is a consequence of neuroendocrine tumors releasing 5-hydroxytryptamine (5-HT) into the systemic circulation, affecting right heart valves, causing fibrosis, and eventually right heart failure. The aim of this study was to determine the effect of valve-replacement on kidney function, liver function, and 5-hydroxyindoleacetic acid (5-HIAA) levels.

**Methods:**

A Retrospective study of 17 patients with CHD who had undergone heart-valve replacement surgery between 2010 and 2019, from the Queen Elizabeth Hospital Birmingham. 5-HIAA levels, liver, and kidney function were measured in addition to hepatic inferior vena cava (IVC) diameter and its relationship to carcinoid symptoms.

**Results:**

Eleven patients were male and six were female. At time of surgery, average age was 66.6 ± 8.1 years and average BMI was 25.8 ± 5.5 Kg/cm^2^. Three out of 17 patients had one valve replaced, 13/17 had two replaced (tricuspid and pulmonary), and 1/17 had three replaced (tricuspid, pulmonary and aortic). There was a 31% average decline in 5-HIAA [799.8 (343.6–1078.0) to 555.3 (275.8–817.9), *p* = 0.011], a 35% decline in bilirubin [20 (16–29) to 13 (10–19), *p* = < 0.001], and a 15% reduction in the short and long axes of the IVC after valve-replacement surgery [20.0 (18.0–25.0) and 36.5 (29.0–39.8) to 17.0 (14.5–19.3) and 31.0 (26.5–34.3) respectively, *p* = < 0.001 and 0.002 respectively].

**Conclusion:**

Valve replacement surgery improves 5-HIAA levels alongside improved liver function and hepatic IVC diameter. These findings are consistent with resolution of congestive hepatopathy, and therefore enhanced clearance of 5-HIAA. This suggests that valve-replacement surgery can indirectly have beneficial outcomes on hepatic function and is also associated with a drop in the circulating levels of tumor derived serotonin.

## Introduction

Carcinoid heart disease (CHD) is a rare condition affecting patients with carcinoid syndrome, which can result in heart failure secondary to the various vasoactive mediators produced by neuroendocrine tumors (1). Serotonin (5-hydroxytryptamine, 5-HT) plays a major role in the development of CHD. 5-HT can bind to 5-HT2B receptors expressed on cardiac valves and myocytes, causing inflammation and fibroblast proliferation, eventually leading to valve fibrosis, retraction of leaflets and valve incompetence (2, 3) typically producing isolated right-sided heart failure (2).

Patients often present late with the signs and symptoms of advanced carcinoid syndrome (cutaneous flushing and diarrhea), CHD and heart failure (4). It is recommended that patients with carcinoid syndrome and/or raised 5-hydroxyindoleacetic acid (5-HIAA, a degradation product of 5-HT) levels have 6–12 monthly N-terminal prohormone of brain natriuretic peptide (NT-proBNP) measurements to screen for CHD (5, 6). Raised NT-proBNP in these patients is a screening tool that, if elevated, mandates the performance of transthoracic echocardiography (TTE) to detect CHD, i.e. heart valve thickening and regurgitation (5).

**Figure 1:**
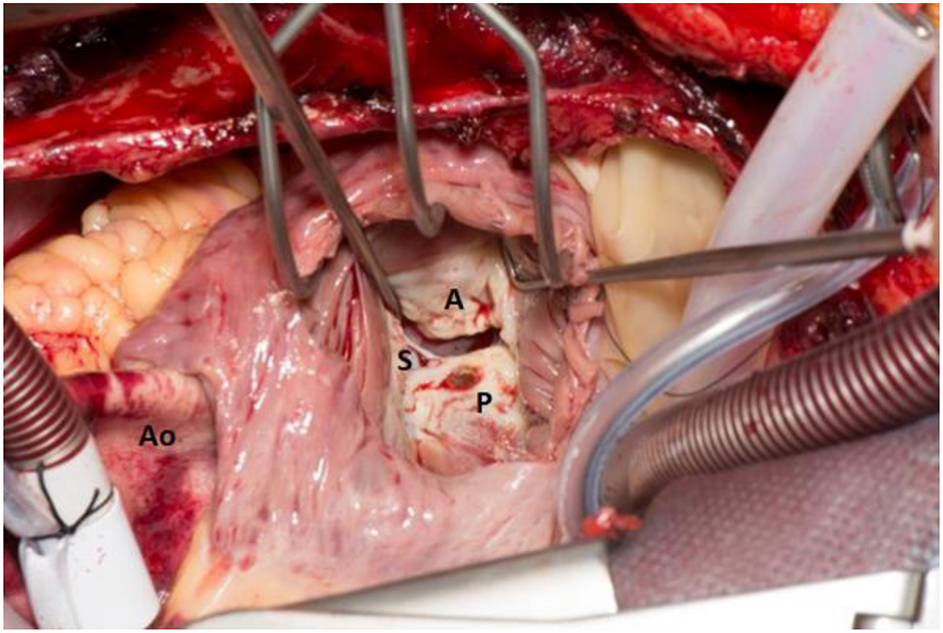
The right atrium is opened and retracted exposing the Tricuspid Valve (TV). The cardiopulmonary bypass caval cannulae can be seen. The Aorta (Ao) is on the left hand side. The TV can be seen through the right atrium and the leaflets are labeled respectively (anterior leaflet- A, posterior leaflet- P and septal leaflet- S). The leaflets are thickened and fibrosed and the septal leaflet is plastered onto the ventricular septum inferiorly.

CHD is treated with a combination of diuretics to reduce fluid overload, and somatostatin analogs to reduce circulating 5-HT levels (4). The definitive treatment for CHD remains heart-valve replacement surgery (see Figure 1), which has been shown to improve performance status; however, it has not yet been proven to improve life expectancy – indeed, surgery itself is associated with a 10–20% peri-operative mortality (although the Mayo clinic reports a figure of 5–6% for their latest cohort) (5, 7, 8). Determining when and on whom to operate remains a matter of debate (7). We previously reported in a retrospective study that valve-replacement surgery was associated with a post-operative reduction in 5-HIAA levels (a marker of tumor activity) which suggests that treating CHD may influence tumor activity. The study was limited by the small number of participants (9). A detailed description of peri-operative management is discussed in our previous paper (10).

The purpose of this study therefore is to expand on these findings and to determine whether surgery diminishes circulating tumor hormones. A secondary aim of the study is to investigate whether surgery improves end organ function, specifically the liver.

## Materials and Methods

### Study Cohort

This is a retrospective, single center study of patients, who underwent valve replacement surgery at the Neuroendocrine Tumor Centre at the Queen Elizabeth Hospital Birmingham. All patients with CHD who underwent heart-valve replacement surgery between 2010 and 2019 (2010 marking the introduction of the “PORTAL” electronic record) were eligible for inclusion (n = 40). Patients were referred for surgery if they met one of the following criteria: stable carcinoid tumor load; severe valvular dysfunction; poor exercise tolerance. Patients were excluded from the study if they died before follow-up tests could be completed (n = 4); did not have complete data, (n = 13); or had concomitant changes to medical management that could confound the 5-HIAA levels, e.g. somatostatin analogs commencement or alteration in dose) or application of interventional radiology treatment (trans-arterial embolization) (n = 6). Conversely, no patients had changes to medical management of heart failure necessitating exclusion.

This left 17 patients within this study.

### Study Measures

All demographic, hematology, biochemical and imaging patient data were drawn from electronic patient records and imaging reports. Within the NET-CHD service, all patients are routinely admitted to hospital for detailed assessment, establishing fitness to proceed with surgery. These admissions were no more than six months from the time of planned surgery.

Renal function was measured using urea and creatinine; liver function: bilirubin, albumin, and PTT; tumor-related hormone activity using 24-h urinary 5-HIAA, with chromogranin-A as a general marker of tumor burden. These data were collected within a range of one to six months either side of surgical intervention.

**Figure 2:**
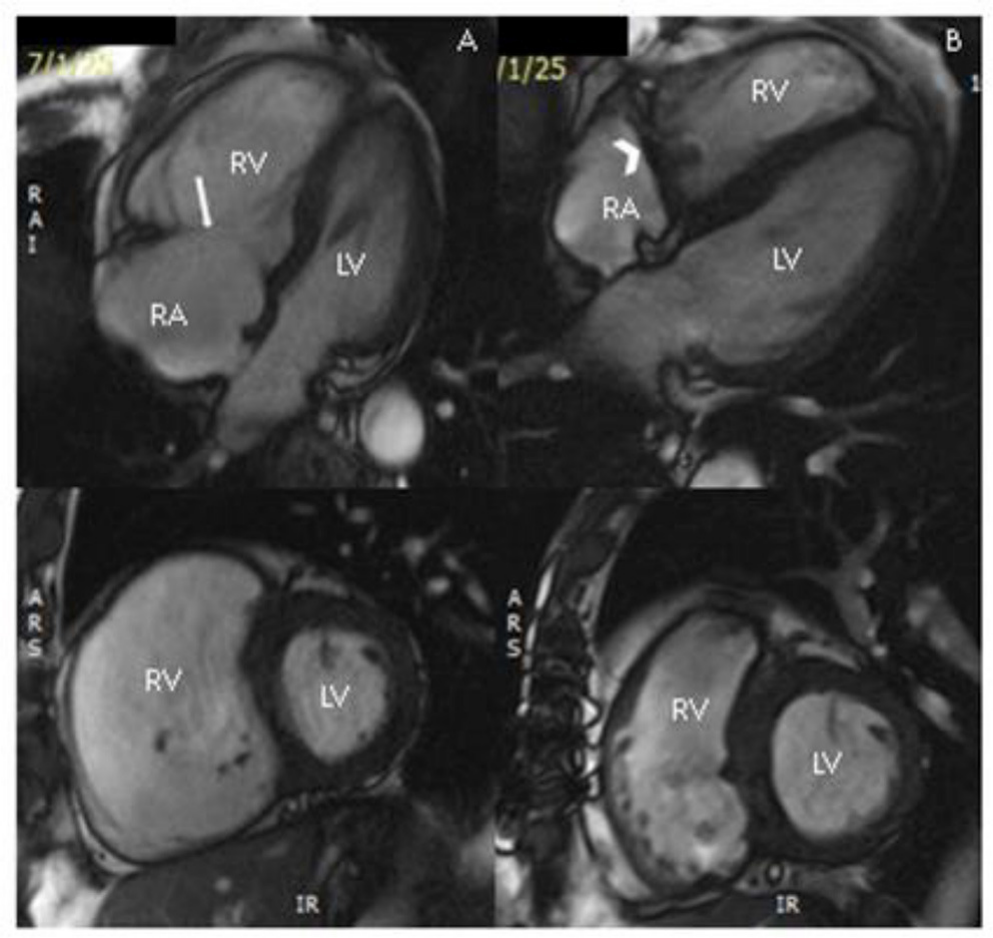
Cardiac magnetic resonance images before surgery **(A)** demonstrating severe right ventricular dilatation and flattening of the inter-ventricular septum, and post-tricuspid valve replacement **(B)** demonstrating significant reduction in right ventricular volume; top panel: still from four-chamber cine sequence; lower panel: still from short-axis cine stack at mid-ventricular level. RV, right ventricle; LV, left ventricle; RA, right atrium; arrow, native tricuspid valve; chevron, tricuspid valve replacement.

**Figure 3:**
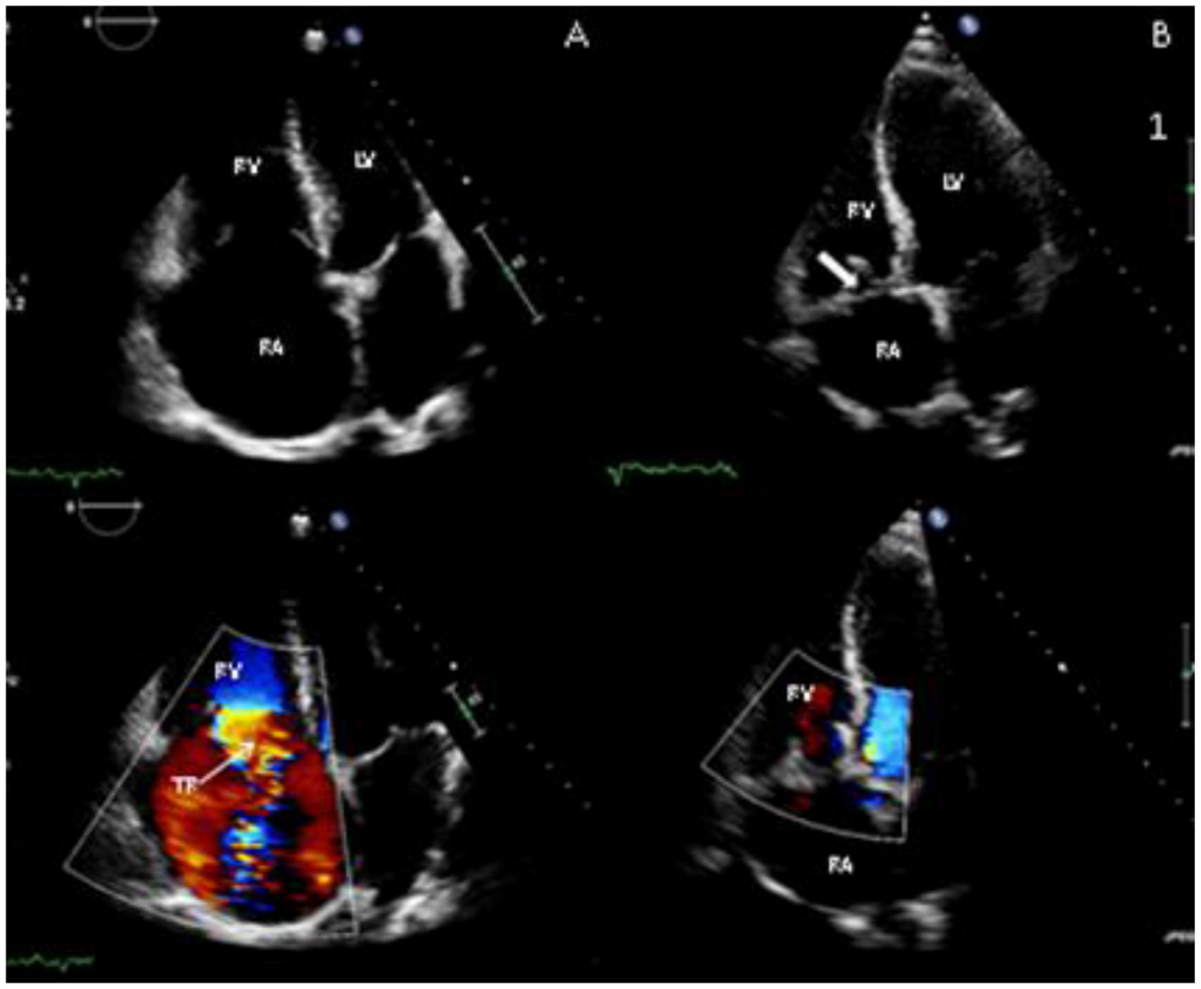
Transthoracic echocardiography images before surgery **(A)** and after surgery **(B)** showing apical 4-chamber view of the tricuspid valve (TV). In the pre-surgery images there is failure of leaflet co-aption (note closed position of mitral valve) due to valve degeneration, with severe tricuspid regurgitation (TR) on colour Doppler (lower panels); post-surgery, the right ventricle (RV) and right atrium (RA) have reduced in size and there is a tricuspid valve bioprosthesis (thick arrow) *in situ*, with resolution of TR.

IVC diameters were measured at the confluence of the hepatic vein with the IVC using routine CT scans that had been performed before and after surgery for the purpose of monitoring cancer progression. Cardiovascular magnetic resonance imaging (CMR) and TTE are routinely performed as part of patient assessment and planning for surgery (see Figures 2, 3).

Carcinoid symptoms were quantified by patient-reported frequency of flushing and diarrhea before and after surgery.

### Data Analysis

Data were tabulated in Microsoft Excel; for each non-parametric measure, the median was calculated. SPSS Statistics (version 23) was used to calculate interquartile ranges, and to apply paired Wilcoxon tests to generate *p*-values. Graphpad Prism 7 was used to generate before and after graphs, which show individual patient data. For parametric data, means were calculated instead; and paired *t*-tests were used to generate *p*-values.

## Results

### Demographics

Of the 17 patients included, 11 were male, six were female. The average age at time of surgery was 66.6 ± 8.1 years. The average BMI at time of surgery was 25.8 ± 5.5 kg/cm^2^.

### Surgical Perspective

Three patients had one valve replaced (tricuspid); 13 had two valves replaced (tricuspid and pulmonary); and one patient had three valves replaced (tricuspid, pulmonary and aortic). All patients received bioprosthetic valves.

The Medtronic Hancock II stented bioprosthesis was implanted in the tricuspid position in 12 patients (sizes 25–29 mm). The St Jude Epic stented bioprosthesis was implanted in the trucuspid position in five patients, and in the pulmonary position in four patients (sizes 21–29 mm). The Edwards Perimount stented bioprosthesis was implanted in the pulmonary position in 10 patients, and in the aortic position for one patient (sizes 21–23 mm).

In addition, three patients underwent a coronary artery bypass graft (CABG) procedure during valve replacement surgery; and four patients underwent a patent foramen ovale (PFO) closure.

The median length of hospital stay for patients discharged after surgery was 15 days (9–34).

Post-operatively, five patients developed tricuspid regurgitation after a median of 95 weeks (32–349 weeks); and six patients developed pulmonary regurgitation after a median of 28 weeks (21–105). One patient developed stenosis of the tricuspid valve, which was detected on TEE at 93 weeks; another patient developed stenosis of the tricuspid and pulmonary valves at 95 weeks.

### Medical Perspective

Sixteen out of 17 patients had small bowel primary; one patient had bronchial primary. Sites of metastasis is documented in Table 1. Sixteen out of 17 patients had metastases to the liver; four to the mesentery; two to bone; and one to mediastinum and retroperitoneum; and one to the pancreas.

**Table 1:** Sites of metastasis.

**Site of metastasis**	**Number of patients**
Liver	16
Mesentery	4
Bone	2
Mediastinum	1
Pancreas	1
Retroperitoneum	1

Tumor load in the liver was determined at NET MDT; mean tumor load = 52.3% ± 18.2 (range 15–80%). All but one had both lobes involved.

At time of surgery, 2/17 patient were on beta blockers; 10/17 were on diuretics; and 17/17 were on somatostatin analogs.

The frequency and severity of carcinoid symptoms were not collected in a sufficiently systematic fashion before and after surgery. Thus, it was not possible to compare them in a meaningful way, and they have therefore not been included in this paper.

### Survival Data

In our patient cohort, 10/17 were deceased at the time of data capture; of these deceased patients, survival ranged from 9 to 413 weeks, with a median survival of 78 weeks.

### Summary of Results

A summary of results can be found in Table 2.

**Table 2:** Summary of markers, before and after surgery.

**Marker**	**Normal range**	**Pre-Treatment**	**Post-Treatment**	***P*-value**
Urea, *mmol/L* (*N* = 16)	2.5-7.8	6 (5–8)	7 (5–8)	0.463
Creatinine, μ*mol/L* (*N* = 16)	64-104	94 (74–120)	94 (68–108)	0.349
Bilirubin, μ*mol/L* (*N* = 15)	<21	20 (16–29)	13 (10–19)	<0.001
Albumin, g/L (*N* = 15)	35–50	43 ± 5	41 ± 5	0.134
INR (*N* = 9)	0.8–1.2	1.2 (1.1–1.3)	1.2 (1.1–1.2)	0.984
24 hr urinary 5-HIAA, μ*mol/L/24 hours* (*N* = 14)	0–45	799.8 (343.6–1078.0)	555.3 (275.8–817.9)	0.011
Chromogranin A, *pmol/L* (*N* = 13)	<60	468.0 (246.5–1586.0)	1366.0 (360.5–2340.0)	0.839
Maximum short axis of the hepatic portion of the IVC, *mm* (*n* = 17)	n/a	20.0 (18.0–25.0)	17.0 (14.5–19.3)	<0.001
Maximum axial diameter of the hepatic portion of the IVC, *mm* (*n* = 17)	n/a	36.5 (29.0–39.8)	31.0 (26.5–34.3)	0.002

### Tumor Markers

**Figure 4:**
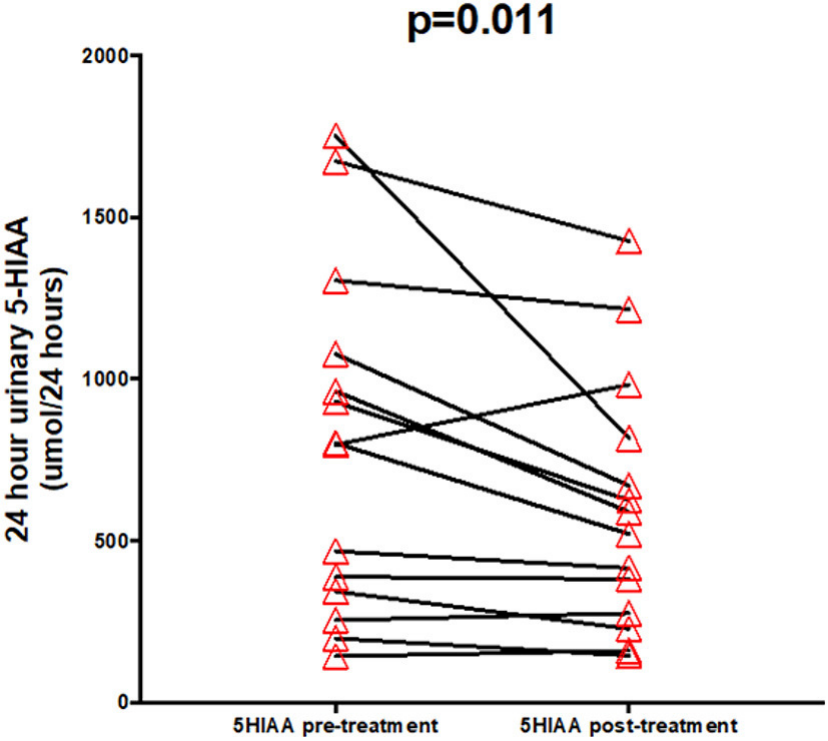
Urinary 5-HIAA pre- and post-treatment.

Twenty four hour urinary 5-HIAA (normal range: 0–45 μmol/24 h) improved by 31% following surgery (*p* = 0.011) from a mean of 799.8 (343.6–1078.0) to 555.3 (275.8–817.9) μmol/24 h. Fourteen of 11 patients had an improvement in 24 h urinary 5-HIAA post-surgery (see Figure 4). Three patients had increase in 5-HIAA levels of 8, 12, and 42%.

**Figure 5:**
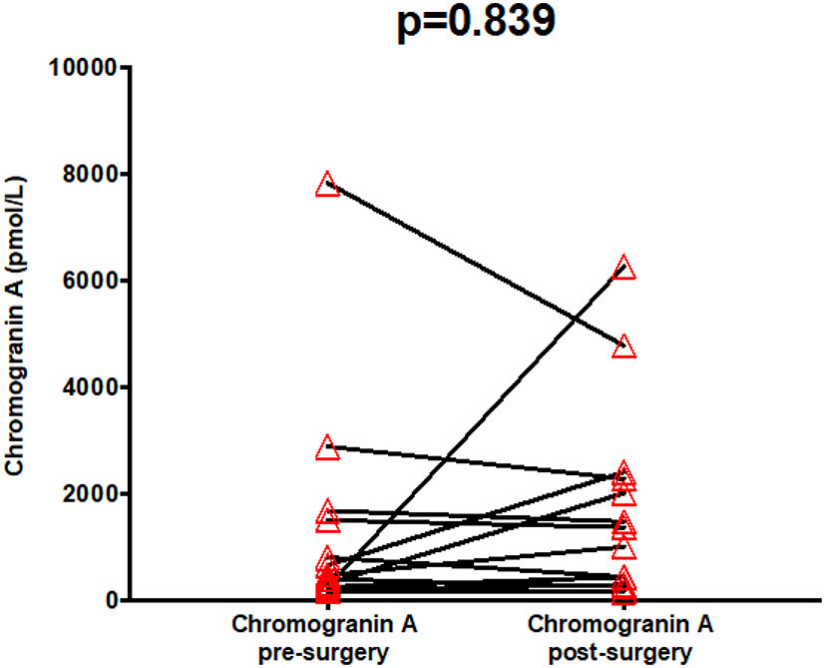
Serum chromogranin A pre- and post-treatment.

Chromogranin A (normal range: <60 pmol/L) did not significantly change after surgery (*p* = 0.839) (see Figure 5).

### Liver Function

The only liver function marker to change significantly (p <0.001) was bilirubin (normal range: <21 μmol/L), which improved from an average of 20 μmol/L (raised) to 13 μmol/L (within normal physiological range), representing a 35% improvement.

The average albumin (normal range: 35–50 g/L) pre-surgery was within normal range (43 *g/L*) and did not significantly improve (*p* = 0.134). Likewise, INR (normal range: 0.8–1.2) did not change on average and remained within normal physiological range (1.2).

**Figure 6:**
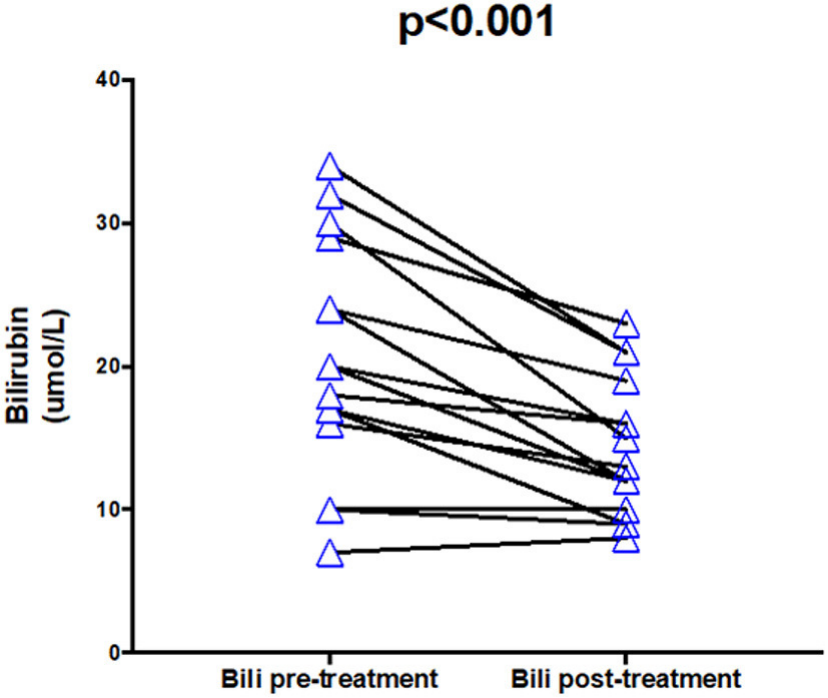
Bilirubin pre- and post-treatment.

Thirteen out of 15 patients had an improvement in bilirubin levels post-surgery (see Figure 6). One patient remained the same, one patient worsened slightly.

### Hepatic IVC Diameter

**Figure 7:**
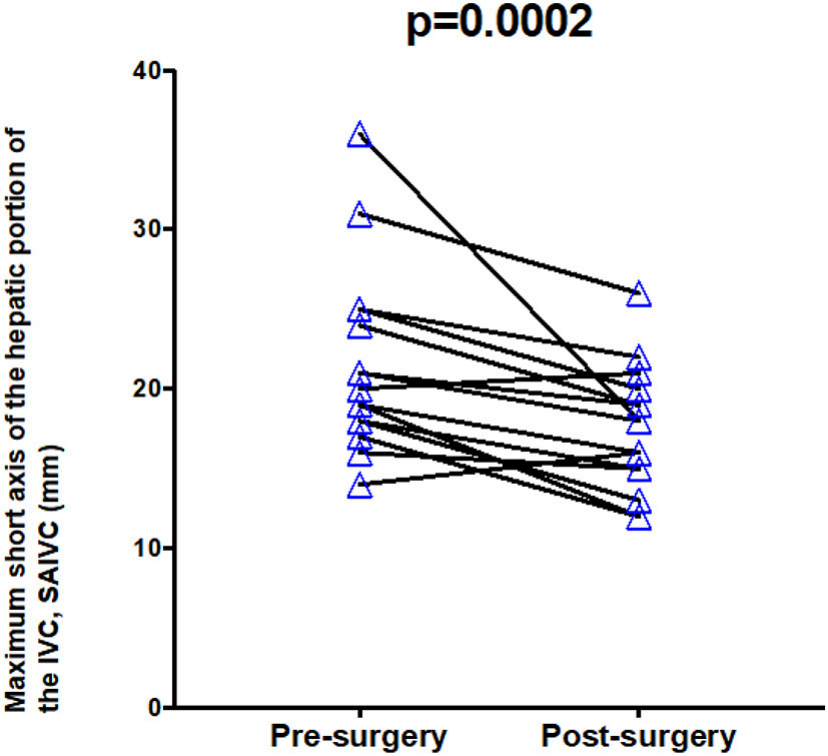
Maximum short axis of hepatic IVC pre- and post-treatment.

**Figure 8:**
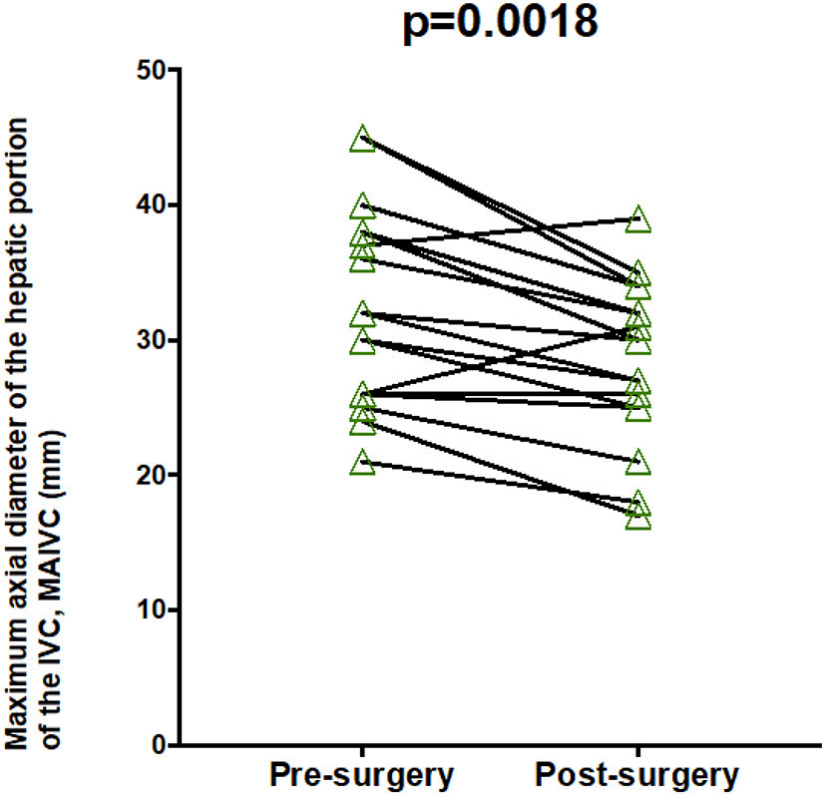
Maximum long axis of hepatic IVC pre- and post-treatment.

Both the short and long axes of the hepatic portion of the IVC were significantly reduced by an average of 15% following surgery (*p* = <0.001, 0.002 respectively). When examined individually, 15/17 patients showed an improvement in both axes of measurement (see Figures 7, 8). In both instances, two patients showed an increase in the axes of measurement.

### Renal Function

There was no significant change in serum urea (normal range: 2.5–7.8 mmol/L) and creatinine (normal range: 64–104 μmol/L) post-surgery (*p* = 0.463, 0.349 respectively) after surgery, and remained within physiological range (6 to 7, 94 to 94 μmol/L respectively).

## Discussion

### Primary Outcomes

For patients with CHD, heart-valve replacement surgery is associated with a reduction in 5-HIAA levels and bilirubin levels. These findings reflect improvement in liver function. These results complement our previous observations showing a reduction in 5-HIAA levels with cardiac surgery (9).

Of the 14/17 patients with complete sets of 5-HIAA measurements, three experienced a rise after surgery; with increases of 8, 12, and 42%, despite a reduction in serum bilirubin levels. However, two out of three of these patients had cancer progression on cross-sectional imaging and rising serum chromogranin-A levels, indicating that a rising 5-HIAA level post cardiac surgery warrants investigation for tumor progression.

Increased liver enzymes (particularly ALP and GGT), bilirubin, and INR with a reduced albumin has been described in right heart failure (11). In our case series with CHD, we noted that other markers of liver function (ALP, GGT, albumin and INR) were within normal physiological ranges before surgery and did not change with surgery. Bilirubin was the only marker that was elevated and showed significant decrease following surgery in line with previous reports in tricuspid regurgitation (11). Bilirubin could therefore serve as a sensitive marker of liver dysfunction in CHD.

Renal function (urea and creatinine) did not significantly change following surgery. Based on these findings, improvement in renal function is unlikely to play a role in the reduction of 5-HIAA levels.

Overall, these findings are consistent with our hypothesis that reduction in 5-HIAA can be attributed to improvement in congestive hepatopathy and hence liver function and enhanced clearance of 5-HIAA.

Both the long and short axes of the hepatic IVC were reduced after surgery, which reflects improvements in the pressure within this vessel.

Right heart overload and regurgitation of blood in to the IVC is well-known to cause what is termed “congestive hepatopathy” as seen in children and young adults with univentricular hearts (12). This creates a constant back pressure on the liver leading to an increase in liver enzymes and bilirubin, liver fibrosis and eventually cirrhosis (12).

A similar mechanism seems to be occurring in patients with tricuspid regurgitation due to CHD. The regurgitation of blood from the right ventricle into the IVC and then hepatic veins can often be seen on CT scan by analyzing the flow of contrast in the blood vessels.

**Figure 9:**
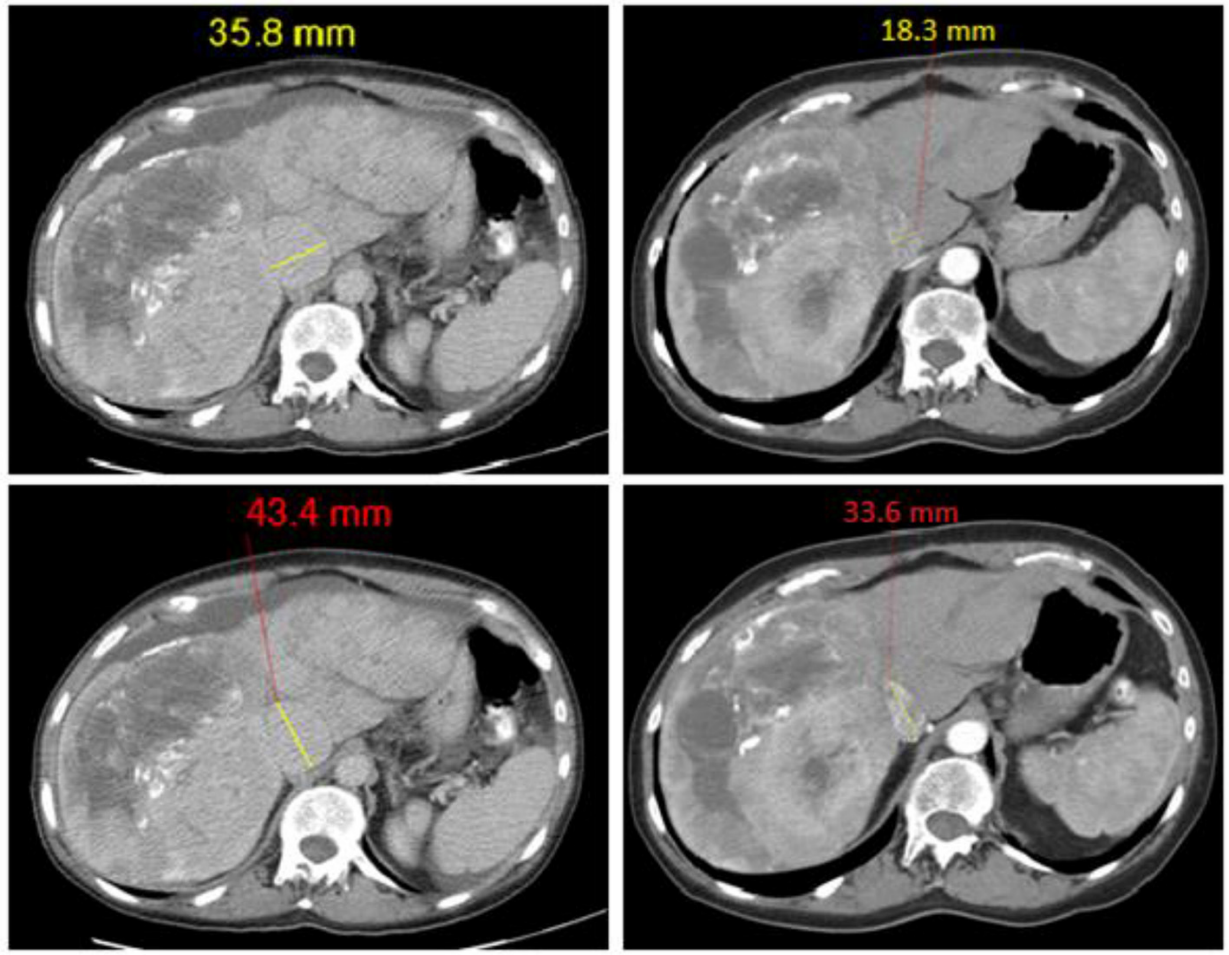
Representative imaging for data in Figures 5, 6 showing reduction in IVC diameters following tricuspid valve replacement; before surgery images on left hand side, after surgery images on right hand side.

Replacement of the tricuspid valve resolves the regurgitation (see Figures 7–9.) and is proposed to be the mechanism for the improvement in serum bilirubin levels (13). This suggests that an approach to managing CHD that prioritizes improving liver function may help to improve 5-HIAA levels and likely patient.

### Limitations

The retrospective study design is inherently less powerful than a prospective study design.

After application of exclusion criteria, 17 out of 40 patient were included in the study, which means that the results may be subject to some selection bias.

It was not possible to accurately quantify the severity of carcinoid syndrome with the existing quality of life questionnaires i.e. QLQ C30 and QLQ GINET21 as they do not capture carcinoid syndrome in terms of frequency or severity.

## Conclusion

Right-sided heart-valve replacement in CHD leads to major physiological changes due to changes in hemodynamics and IVC pressures brought about by right heart failure and regurgitation into the inferior vena cava.

Here we present novel data showing for the first time an improvement in serum 5-HIAA levels, a marker strongly related to carcinoid syndrome symptoms and CHD, in a cohort of patients where confounding variables have been excluded.

Furthermore, we present evidence for the first time that improvement in 5-HIAA levels occur in parallel with an improvement in liver function, namely a significant reduction in bilirubin (which appears to be a more sensitive marker of liver dysfunction). We demonstrate that after valvular surgery the raised IVC pressure from right heart failure improves as evidenced by a significant reduction in IVC diameters. Reduction in IVC diameter and bilirubin levels suggest resolution of congestive hepatopathy and hence a reduction in 5-HIAA levels. In addition to the improvement in cardiac function following valvular repair in CHD, our results demonstrate that there are additional benefits leading to improved liver function and reduction in 5-HT levels and thus may improve the options for additional tumor directed therapies in these patients.

## Data Availability Statement

The authors confirm that the data supporting the findings of this study are available within the article. Raw data that support the findings of this study are available from the corresponding author, upon reasonable request.

## Ethics Statement

The studies involving human participants were reviewed and approved by Clinical Audits and Registries Management Service (CARMS). Written informed consent for participation was not required for this study in accordance with the national legislation and the institutional requirements.

## Author Contributions

Conception and design: HS and TS. Collection and assembly of data: HS. Data analysis and interpretation: HS, VS, and TS. All authors contributed in manuscript writing and final approval of manuscript.

## Funding

Funding was provided by University Hospitals Birmingham Neuroendocrine Tumor Fund.

## Conflict of Interest

The authors declare that the research was conducted in the absence of any commercial or financial relationships that could be construed as a potential conflict of interest.

## Publisher's Note

All claims expressed in this article are solely those of the authors and do not necessarily represent those of their affiliated organizations, or those of the publisher, the editors and the reviewers. Any product that may be evaluated in this article, or claim that may be made by its manufacturer, is not guaranteed or endorsed by the publisher.
